# Kinetics of HE4 and CA125 as prognosis biomarkers during neoadjuvant chemotherapy in advanced epithelial ovarian cancer

**DOI:** 10.1186/s13048-021-00845-6

**Published:** 2021-07-19

**Authors:** Jorge A. Alegría-Baños, José C. Jiménez-López, Arely Vergara-Castañeda, David F. Cantú de León, Alejandro Mohar-Betancourt, Delia Pérez-Montiel, Gisela Sánchez-Domínguez, Mariana García-Villarejo, César Olivares-Pérez, Ángel Hernández-Constantino, Acitlalin González-Santiago, Miguel Clara-Altamirano, Liz Arela-Quispe, Diddier Prada-Ortega

**Affiliations:** 1 Oncology Center, Médica Sur, Mexico City, Mexico; 2 Chemical Sciences Faculty, Universidad La Salle, Benjamín Franklin 45, 06140 Mexico City, Mexico; 3 Science Faculty, Universidad Nacional Autónoma de México (UNAM), Mexico City, Mexico; 4 Instituto Nacional de Cancerología, Colonia Seccion XVI, San Fernando 22, 14080 Tlalpan, Mexico City, Mexico; 5 Centro Médico Nacional Siglo XXI, Mexico City, Mexico; 6 Support and Promotion Program for Student Research, UNAM, Mexico City, Mexico; 7 Department of Skin and Soft Tissue, Instituto Nacional de Cancerología, Mexico City, Mexico; 8 Department of Molecular Imaging, Instituto Nacional de Cancerología, Mexico City, Mexico; 9 Department of Environmental Health Sciences, Mailman School of Public Health, Columbia University, 722 W 168th St, New York, NY 10032 USA

**Keywords:** Biomarkers kinetic, HE4, CA125, Gynecological cancer, Ovarian cancer, Neoadjuvant chemotherapy

## Abstract

**Background:**

Ovarian cancer (OC) is considered the most lethal gynecological cancer, of which more than 65% cases are diagnosed in advanced stages, requiring platinum-based neoadjuvant chemotherapy (NACT).

**Methods:**

A prospective-longitudinal study was conducted among women with advanced epithelial ovarian cancer (AEOC), III and IV stages, and treated with NACT, at the National Cancer Institute – Mexico, from July 2017 to July 2018. Serum samples were obtained for quantification of CA125 and HE4 using ELISA at the first and in each of the three NACT cycles. The therapeutic response was evaluated through standard tomography. We determined whether CA125 and HE4, alone or in combination, were associated with TR to NACT during follow up.

**Results:**

53 patients aged 38 to 79 years were included, 92.4% presented papillary serous subtype OC. Higher serum HE4 levels were observed in patients with non-tomographic response (6.89 *vs* 5.19 pmol/mL; *p* = 0.031), specially during the second (*p* = 0.039) and third cycle of NACT (*p* = 0.031). Multivariate-adjusted models showed an association between HE4 levels and TR, from the second treatment cycle (*p* = 0.042) to the third cycle (*p* = 0.033). Changes from baseline HE4 levels during the first cycle was negative associated with TR. No associations were found between CA125 and TR.

**Conclusions:**

Serum HE4 levels were independently associated with TR among patients with AOEC treated with NACT, also a reduction between baseline HE4 and first chemotherapy levels was also independently associated with the TR. These findings might be relevant for predicting a lack of response to treatment.

**Supplementary Information:**

The online version contains supplementary material available at 10.1186/s13048-021-00845-6.

## Introduction

Ovarian cancer (OC) is the second most common gynecological neoplasm, behind endometrial cancer, and is the most lethal gynecological cancer, being the eighth leading cause of cancer death in women [1]. Almost 80% of cases are diagnosed as advanced stage disease. Current treatment for advanced epithelial ovarian cancer (AOEC) involves primary debulking surgery followed by a adjuvant chemotherapy regimen based on the combination of platinum and taxane, or the initial administration of neoadjuvant chemotherapy (NACT) followed by surgery [2]. The absence of residual disease in primary surgery is one of the most relevant prognostic factors for overall survival [3, 4]. NACT and primary debulking surgery have the same efficacy when used at their maximal possibilities, but the toxicity profile is different. NACT is a treatment regimen that can be considered in selected patients [5–7], it does not negatively affect survival compared to primary debulking surgery plus postoperative chemotherapy and has even shown a significant reduction in perioperative complications and mortality [8, 9].

Tumor biomarkers are clinical or biological characteristics that are qualitatively or quantitatively modified because of a malignant neoplastic condition, being detectable in tissue or fluids [10] as prognosis tools, predictive markers for clinical efficacy, and therapeutic response assessment [11, 12]. The tumor biomarker carbohydrate antigen 125 (CA125) is a high molecular weight glycoprotein (> 200 kD), expressed as a membrane-bund protein at the surface of the coelomic epithelium during embryonic development, and of cells that undergo metaplastic differentiation into a Müllerian-type epithelium [13]. Elevated serum CA125 levels can be detected under physiological circumstances (e.g., pregnancy), in benign gynecological and non-gynecological diseases, also in several malignant entities (e.g., ovarian, endometrial, breast, and colon cancers) [13, 14]. With a cut-off serum level of 35 U/mL, CA125 shows elevated values in 75% to 90% of patients with advanced ovarian cancer. It has a recognized role in the OC diagnosis, follow-up, treatment response assessment, and recurrence detection [15–17]. The CA125 has a sensitivity of 71% to 78% and a specificity of 75% to 94% for ovarian cancer diagnosis.

Some studies have analyzed the predictive value of CA125, based on the level reached at the end of NACT, in patients with ovarian epithelial cancer, yielding different cutoff points [18–21]. A reduction in CA125 to less than 65 IU/mL, or a reduction greater than 50% from baseline, before neoadjuvant chemotherapy, was an independent prognostic factor for survival [22].

Human Epididymal Protein 4 (HE4) is a 13 kD protein (20–25 kD in its mature glycosylated form); it belongs to the family of WAP-type four-disulfide core (WFDC), a group with potential trypsin inhibitory properties [23–26]. It has been proposed as a proteinase inhibitor with utility in the pulmonary immune system and the sperm maturation process, and it is expressed at low concentrations in various healthy tissues, including the respiratory and reproductive epithelium [27]. HE4 is overexpressed in most subtypes of ovarian epithelial cancer [28] and some adenocarcinomas such as lung, endometrium and breast [29–32].

About its role in carcinogenesis, HE4 has been involved in cell cycle regulation and tumor cell proliferation, noting that the silencing of the HE4 gene results in cell cycle arrest in the G0/G1 phase and blocking the progression from the G1 phase to the synthesis or S phase [33, 34]. HE4 is overexpressed in ovarian carcinomas, with a cut-off serum level of 70 pmol/L with higher specificity (95%) than CA125 [35]. HE4 can also be detected in urine with a specificity of 94.4% [36].

The exploration of the prognosis and predictive value of the combination of the biomarkers CA125 and HE4 in OC remains controversial [37–40]. In addition, several methodologies have been reported to assess tumor biomarker kinetics during OC treatment. Almufti et al., [41], classified the strategies and approaches to evaluate the prognostic and predictive utility of tumor biomarkers, based on the number of quantifications performed, which could be studies with a single measure or trials with two or more quantifications. over time, called kinetic studies. In the case of CA125 and HE4, their kinetics during NACT in OC has not been sufficiently investigated.

Finally, as a main objective, we set out to determine the kinetics of serological CA125 and HE4 during CO treatment and their associations with the tomographic response to platinum and taxane therapy in Mexican patients receiving neoadjuvant chemotherapy for advanced epithelial ovarian cancer.

## Materials and methods

A longitudinal study was carried out, including women who attended the National Cancerology Institute—Mexico (NCI-Mx), from July 2017 to July 2018, age > 18 years, with a diagnosis of epithelial ovarian cancer, with advanced disease, stages clinical III or IV from the International Federation of Gynecology and Obstetrics (FIGO) classification, candidates for treatment with neoadjuvant chemotherapy with carboplatin (area under the curve 5–6) and paclitaxel (175 mg / m2), every three weeks. Patients with a double primary tumor, mucinous histology, and those with previous cancer treatment (surgical or systemic) were excluded.

Serum samples were obtained for CA125 and HE4 quantification at the beginning of chemotherapy (baseline) and during every one of the three neoadjuvant treatment cycles. An evaluation of the therapeutic response was subsequently performed by the tomographic standard using the following criteria: “With a response”, including complete and partial response,

or “No response”, according to Response Evaluation Criteria In Solid Tumors (RECIST 1.1).

The association between the CA125 and HE4 kinetics (absolute value and delta of change between the assessments) of biomarkers and the response tomographic was evaluated. We grouped tomographic response in " favorable response", which included full or partial response, and "no response", which included stable disease and disease progression. The serum HE4 and CA125 quantification were performed using the ARCHITECT HE4 assay by a method of Chemiluminescence Microparticle Immunoassay (CMIA).

To compare the values of the two biomarkers, normality was analyzed with histogram, Shapiro–Wilk, and Kolmogorov–Smirnov tests, observing the non-normality distribution of the data, thus a logarithmic transformation of the values was performed to be evaluated. Descriptive and inferential tests, including correlations and a multivariate analysis adjusting by age, basal ECOG, smoking and clinical staging of the FIGO classification were performed.

## Results

**Fig. 1:**
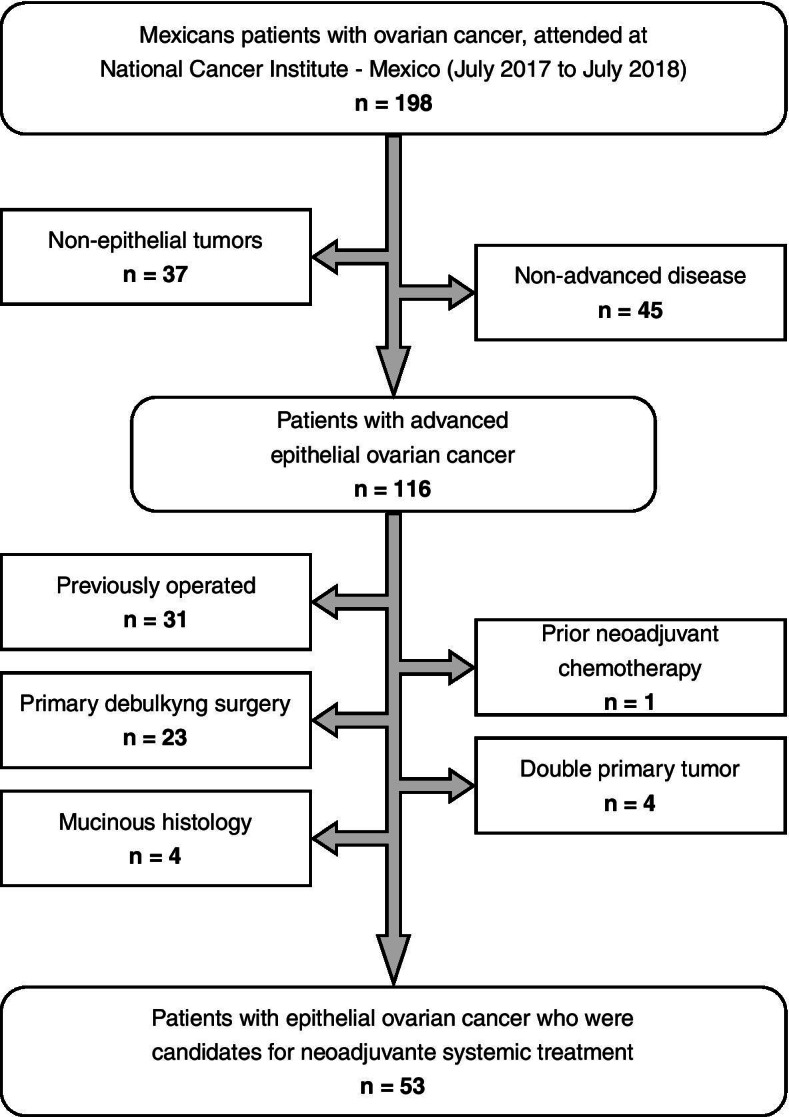
Flowchart sample selection

One hundred ninety-eight patients with ovarian cancer were initially included. From them, 116 patients (58.9%) showed epithelial histological subtype and advanced stage of the disease. Only 53 patients (26.9%) met the inclusion criteria for participation in the study. A previous surgical intervention (15.6%) and a primary cytoreduction (11.6%) were the most frequent causes of exclusion criteria (Fig. 1).

Of the 53 patients evaluated, we found a mean age of 57.8 years (Standard deviation [SD] 10.3 years). In this group, 43.4% was overweight, and 34.0% had high blood pressure. At diagnosis, the main symptoms were creased abdominal girth 75.5% and diffuse abdominal pain (66.6%). The most frequent histopathological subtype was the papillary serous (92.4%), and the majority (52.8%) were in the clinical stage IVB.

The variable "tomographic response" was grouped into two categories: “with a response”, which included full or partial response, those were 32 patients (60.4%), and “no response”, which included stable disease and disease progression, 21 patients (39.6%).

The only variable that showed a statistically significant difference between both groups was the histological subtype. It was observed a significantly higher proportion (*p* = 0.044) of papillary serous cases (32 of 49 cases, 65.3%) in the group with responses, vs 17 of 49 cases (34.7%) in the group with no tomographic response, as well as the absence of endometrial subtypes and clear cells in the group with answer (Table 1).

**Table 1:** Clinicopathological characteristics of the patients according to tomographic response (n= 53)

**Variable**	**Category**	**With response (%)**	**Non response (%)**	*p*^a^
Body mass index (WHO)	Overweight	12 (37.5)	11 (52.4)	0.934
	Normal	13 (40.6)	6 (28.6)	
	Obese	7 (21.9)	4 (19.0)	
Diabetes mellitus	Yes	4 (12.5)	2 (9.5)	1.000
	No	28 (87.5)	19 (90.5)	
Systemic Arterial Hypertension	Yes	10 (31.2)	7 (33.3)	1.000
	No	22 (68.8)	14 (66.7)	
Smoking	Yes	6 (18.7)	2 (9.5)	0.335
	No	26 (81.3)	19 (90.5)	
ECOG	0-1	29 (90.6)	20 (95.2)	0.065
	≥ 2	3 (9.4)	1 (4.8)	
Histological subtype	Papillary serous	32 (100)	17 (81.0)	**0.037**
	Endometrioid	0 (0.0)	3 (14.2)	
	Clear cell	0 (0.0)	1 (4.8)	
Clinical stage (FIGO)	IVB	18 (56.3)	10 (47.6)	0.914
	IVA	7 (21.9)	9 (42.9)	
	IIIC	5 (15.6)	2 (9.5)	
	IIIB	1 (3.1)	0 (0.0)	
	IIIA	1 (3.1)	0 (0.0)	

Data is presented as n (%)

^a^ X^2^ tests

Those p values with statistical significance (less than 0.05) are marked in bold

The mean baseline HE4 was 2150.9 pmol / mL (range: 61.5—25,596.3) and that of CA125 was 5367.2 IU / mL (range: 28.2—36,262.4). The mean of both biomarkers decreased in each of the three cycles, with the greatest decline slope after the first cycle (Supplementary Table S1 and Fig. S1).

It was observed that patients with no tomographic response presented a higher level of HE4 [average of 6.19 (95% CI, 4.92–6.84) pmol / mL] compared to the group that showed tomographic response [average of 5.89 (95% CI, 5.65–6.78) pmol / mL], with statistically significant difference (*p* = 0.031), this observation remains independently regardless the cycle where the sample was taken.

**Fig. 2:**
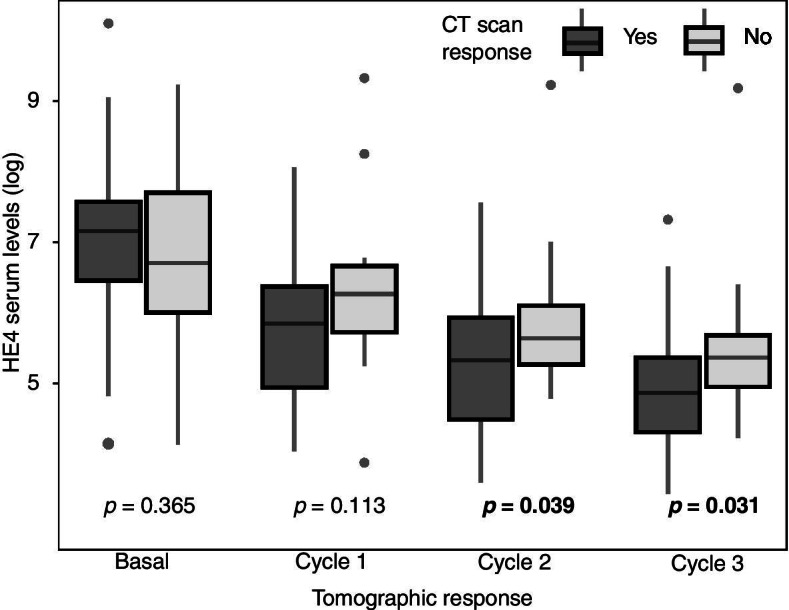
HE4 levels according to tomographic responses and treatment cycle (n = 53). Comparisons between groups according tomographic response are reported using Two Sample T-Test (Welch's T-test). Those p values with statistical significance (less than 0.05) are marked in bold

By analysing the association between basal HE4 serum levels and tomographic response, a higher concentration was found in patients with tomographic response group [7.13 (CI 95%, 6.95–7.31) pmol/mL vs 6.84 (CI 95%, 6.64–7.04) pmol/mL] with no statistically significant difference (Table 2). In each one of the treatment cycles, HE4 values were lower in tomographic response group with statistically significant difference for the second [5.32 (95% CI, 5.17–5.47) pmol / mL versus 5.93 (95% CI, 5.74–6.12) pmol / ml; *p* = 0.039] and third cycle [4.91 (95% CI, 4.79–5.03) pmol / mL versus 5.56 (95% CI, 5.44–5.68) pmol / mL; *p* = 0.031] of treatment (Fig. 2).

**Table 2:** Association between HE4 levels ^a^ and tomographic response (n = 53)

	**Univariable**			**Multivariable**^**b**^		
**HE4**	**Estimated**	**CI 95%**	***p***	**Estimated**	**CI 95%**	***p***
Basal	-0244	-0.689, 0.151	0.357	-0.262	-0.832, 0.308	0.367
To the first cycle	0.444	-0-249, 0.561	0.119	0.524	-0.077, 1.125	0.088
To the second cycle	0.676	0.061, 1.081	0.054	0.752	0.028, 1.475	**0.044**
To the third cycle	0.766	0.175, 1.181	**0.042**	0.843	0.068, 1.617	**0.033**
All values	0.241	0.012, 0.352	**0.038**	0.268	0.030, 0.505	**0.027**

*CI *confidence interval

^a^ Transformed by decimal logarithm. Measurements of all cycles and basal values were included

^b^ Adjusted for age at diagnosis, baseline ECOG, smoking the histological subtype and FIGO stage

Those p values with statistical significance (less than 0.05) are marked in bold

By doing the multivariate analysis with an adjustment by age, basal ECOG, smoking and clinical staging of the FIGO classification, an association statistically significant was observed between HE4 levels and tomographic response, from the second cycle of treatment (*p* = 0.042), remaining until the third cycle (*p* = 0.033). By analyzing all values (basal and each cycle of chemotherapy), statistical significance was observed from univariate and multivariate analysis (Table 2).

Odds Ratio calculation was performed using the applicable variables, taking as response variable the result shown in the tomography after three cycles of chemotherapy. No significant value was found in the univariate analysis, and there was no multivariate model development (Supplementary Fig. S2).

**Fig. 3:**
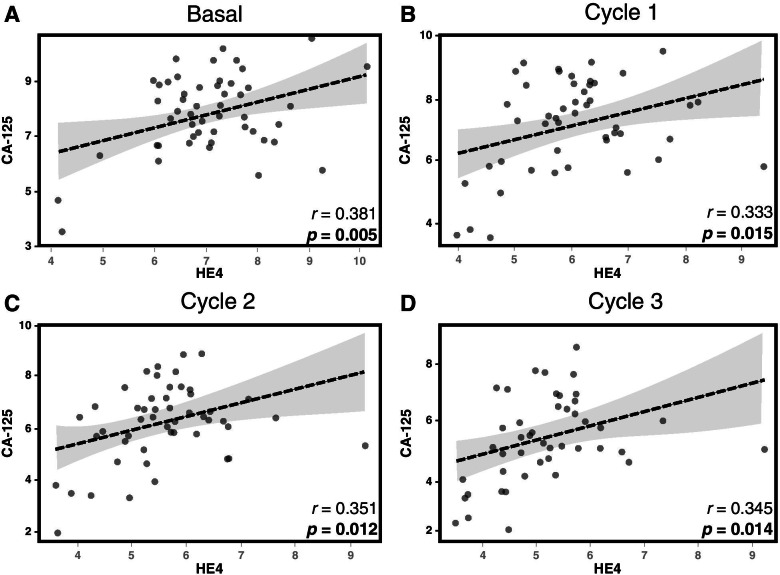
Correlation between serological levels of HE4 and CA125 biomarkers during the different cycles (n= 53).Pearson's correlations shown in **A**, **B**, **C** and **D** are statistically significative with a p-value < 0.05

The analysis of the values up to the sixth cycle, shows that the described behavior was also verified by comparing the difference values in serum levels with respect to the basal level. The data show that in patients with response this decrease is greater (Estimated Effect of -2.01 until the third cycle) compared with patients who showed no tomographic response (Estimated effect of -1.19 until the third cycle). The correlation between the serum levels of the HE4 and CA125 biomarkers was explored, demonstrating collinearity, with similar trends at different times of treatment (Fig. 3).

The association between tomographic response and the difference (delta) between HE4 levels in different treatment cycles was analyzed, and we observed a statistically significant relationship between the difference in basal serum levels and those obtained after the first cycle of chemotherapy, both in the univariate analysis and in the age-adjusted multivariate model at diagnosis, basal ECOG, smoking and FIGO stage (Table 3). Thus, it was shown that a greater reduction between basal HE4 levels and those of the first cycle of chemotherapy was independently associated with the tomographic response. No statistically significant relationship was observed with any other time comparison.

**Table 3:** Association between tomographic response and the difference (delta) between HE4^a^levels (n=53)

	**Univariate**			**Multivariate**^*b*^		
**HE4**	**Est**	**CI 95%**	***p***	**Est**	**CI 95%**	***p***
Between basal and first cycle	0.182	0.033, 0.330	**0.017**	0.194	0.019, 0.368	**0.030**
Between basal and second cycle	0.116	-0.013, 0.245	0.080	0.128	-0.013, 0.269	0.077
Between basal and third cycle	0.090	0.060, 0.240	0.244	0.108	-0.058, 0.274	0.203
Between first and second cycle	0.069	-0.125, 0.263	0.485	0.203	-0.092, 0.498	0.179

*CI* confidence interval

^a^ Transformed by decimal logarithm

^b^ Adjusted for age at diagnosis, basal ECOG, smoking, histological subtype and FIGO stage

Those p values with statistical significance (less than 0.05) are marked in bold

The most frequent adverse event associated with chemotherapy was neutropenia, present in 39 patients (73.6%), being grade 3 or higher in 20 patients (37.6%). The remaining toxicities were nausea in 24 patients (45.3%), anemia in 22 (41.5%), peripheral neuropathy in 16 (30.2), emesis in 12 (22.6%), thrombocytopenia in 9 (17.0), diarrhea in 6 (11.3%), and nephrotoxicity and hepatotoxicity in one patient (1.9%), without showing a statistically significant distribution in relation to the tomographic response (Table 4).

**Table 4:** Adverse events due to chemotherapy according to tomographic response to neoadjuvant chemotherapy (n = 53). Percentage values are calculated within groups

**Adverse event (CTCAE 5.0)**		**With response (%)**	**Without response (%)**	***p-value***^a^
Neutropenia	Grade 0	10 (31.3)	4 (19.0)	0.582
	Grade 1	1 (3.1)	2 (9.52)	
	Grade 2	8 (25.0)	8 (38.1)	
	Grade 3	11 (34.4)	5 (23.9)	
	Grade 4	2 (6.3)	2 (9.5)	
Anemia	Grade 0	19 (35.8)	12 (22.6)	0.773
	Grade 1	5 (9.4)	2 (3.8)	
	Grade 2	7 (13.2)	5 (9.4)	
	Grade 3	1 (1.9)	2 (3.8)	
Thrombocytopenia	Grade 0	29 (90.6)	15 (71.42)	0.134
	Grade 1	2 (6.2)	1 (4.8)	
	Grade 2	0 (0.0)	3 (14.3)	
	Grade 3	1 (3.1)	2 (9.5)	
Nausea	Grade 0	19 (57.6)	11 (52.4)	0.953
	Grade 1	9 (27.3)	7 (33.3)	
	Grade 2	4 (12.1)	3 (14.3)	
	Grade 3	1 (3.0)	0 (0.0)	
Emesis	Grade 0	26 (81.2)	15 (71.4)	0.676
	Grade 1	4 (12.5)	5 (23.8)	
	Grade 2	2 (6.3)	1 (4.8)	
Diarrhea	Grade 0	27 (84.4)	20 (95.2)	0.781
	Grade 1	4 (12.5)	1 (4.8)	
	Grade 2	1 (3.1)	0 (0.0)	
Renal toxicity	Yes	1 (3.1)	0 (0.0)	1
	No	31 (96.9)	21 (100.0)	
Liver Toxicity	Yes	1 (3.1)	0 (0.0)	0.413
	No	31 (96.9)	21 (100.0)	
Peripheral	Grade 0	23 (71.9)	14 (66.7)	0.908
Neuropathy	Grade 1	5 (15.6)	3 (14.3)	
	Grade 2	4 (12.5)	4 (19.0)	

^a^ Fisher exact test; CTCAE 5.0: Common Terminology Criteria for Adverse Events version 5.0

## Discussion

Ovarian cancer is the most lethal gynecological neoplasm in the world, due to its late diagnosis and non-specific symptoms. Within the study, 198 patients with ovarian cancer were evaluated, of which 153 (77.2%) presented advanced stage disease at the time of diagnosis (including epithelial and non-epithelial tumors), which is in line with what is reported in the international literature, where it has been indicated that more than 65% of patients are diagnosed in advanced stages [1]. The average age of presentation of the 53 patients included was 57.8 years (SD ± 10.3 years), lower than that reported globally, being 62 years [1, 42]. This early presentation has been reported in other neoplastic diseases within the Mexican population, highlighting colorectal and prostate cancer.

The main documented comorbidities were overweight (45.2%) and systemic arterial hypertension (43%). The main symptoms were abdominal and nonspecific, in accordance with the literature [43], highlighting the increase in abdominal girth in 40 patients (75.5%) and diffuse abdominal pain in 35 (66.6%).

The only variable that showed statistically significant difference between both groups was the histological subtype, where patients with endometrioid and clear cells subtypes did not have favorable tomographic response with neoadjuvant treatment. Clear cell and mucinous subtypes in advanced disease are associated with a very poor prognosis and resistance to standard treatment [44, 45], however, this observation is limited in our study due to the small sample size (only one case with clear cell histology) and not including mucinous tumors because of their low production of HE4 biomarker.

Adverse events from chemotherapy occurred more frequently at the hematological and gastrointestinal levels, in accordance with the toxicity profile of the platinum-taxane combination reported in the literature. Some other frequent manifestations such as dysgeusia and headache were not reported during medical visits, in up to 11.8% and 7.1% of cases, respectively.

Regarding the biomarker HE4, it has demonstrated protease activity and participation in cell signalling, acting on processes of adhesion, migration, and promotion of tumour growth [46, 47]. In addition to its standard use, approved for the differential diagnosis of malignant adnexal tumors (Risk of Ovarian Malignancy Algorithm), there are assays that explore the value of HE4 as a prognostic marker [48], a predictor of optimal cytoreduction [49], and a potential tool for early diagnosis of recurrence, even better than CA125 [50, 51].

Most of the reports published to date are retrospective. Studies that relate the role of HE4 to the response to chemotherapy often address the adjuvant setting, following primary cytoreduction. In addition, the authors have used various trials (ARCHITECT by Abott®, ELECSYS by Roche®, or EIA by Fujirebio®) and various statistical methods and approaches for data analysis (percentage decrease, low area to Curve, negativization, etc.), which increase heterogeneity and limit the comparison of results.

The pioneering study, which serves to contrast the results of the work carried out in this investigation, was published by Vallius and cols. [52], where 25 patients with advanced epithelial ovarian cancer, treated with neoadjuvant chemotherapy, were evaluated, quantifying the CA125 and HE4 biomarkers in a basal manner and on a later occasion, after completion of adjuvant chemotherapy, prior to interval cytoreduction. The percentage changes of the biomarkers were compared according to the tomographic response and the surgical outcome, concluding that neither CA125 nor HE4 changes were correlated with the tomographic response, observing a clear reduction in serum levels of both biomarkers in all groups, regardless of the radiological response. In fact, patients with disease progression had an average decrease in CA125 levels of around 83%. This was surprising, since in the pilot study with 11 patients previously conducted, a relationship was observed between changes in serum concentration of biomarkers and the tomographic response.

Despite this finding, the report by Vallius and cols. described a favorable relationship between HE4 decline (> 80%) and prognosis in terms of overall survival, with a median of 3.38 versus 1.60 years (*p* = 0.01). This put into question the discordance between survival results and radiological therapeutic response. The authors argued that tomographic guidelines may be a limitation in the optimal assessment of the response to chemotherapy, suggesting to study, the possibility of using functional imaging as PET-CT to optimize the assessment. In addition to the small number of patients, a limitation of the study by Vallius and cols. was to have only two measurements for each patient.

Chudecka and cols. [53], published a study involving 90 patients with ovarian cancer, 42 of whom were treated with neoadjuvant chemotherapy, with HE4 and CA125 measurements taken at diagnosis, after chemotherapy, and before interval cytoreductive surgery, although in the final analysis, the authors only considered the third cycle of chemotherapy.

Preoperative HE4 levels were a predictor of platinum sensitivity (*p-value* = 0.035) and progression-free survival (*p-value* = 0.0492) when normalized or reduced by 50%, but, unlike the reports of Vallius and cols. [52], in multivariate analysis, normalized HE4 levels after chemotherapy (HR = 0.08, *p-value* = 0.0003) or with 50% reduction before interval debulking (HR = 0.39, *p-value* = 0.0496), correlated with improvement in 2 years overall survival.

The present study is original in its design and tries to optimize the limitations of the reported studies previously. It only includes patients with advanced epithelial ovarian cancer treated with neoadjuvant chemotherapy, excluding the group of patients treated with primary cytoreductive therapy and those who have undergone incomplete surgery (oophorectomy, lumpectomy) prior to the start of systemic treatment, due to the alteration that this procedure conditions in the serum level of the biomarkers. In addition, baseline quantifications have been performed and in each cycle of systemic treatment, including all measurements within the final statistical analysis.

Unlike what was reported by Vallius and cols. [52], in this study a statistically significant association was observed between HE4 changes and the tomographic response in patients with epithelial ovarian cancer treated with NACT. These observations highlight the need to increase the sample size (11 and 25 patients, respectively, in the pilot and the trial by Vallius and cols.; 42 patients in the study by Chudecka and cols., standardize the number and timing of the quantifications, as well as the inclusion criteria, homogenizing the type of chemotherapy to be studied (neoadjuvant versus adjuvant) and analyzing both biomarkers to determine the collinearity of the results.

The main findings of our study are that HE4 levels, especially during the second and third cycle, are independently associated with the tomographic response in patients with advanced epithelial ovarian cancer treated with neoadjuvant chemotherapy. Also, a greater reduction between the basal HE4 levels and those of the first cycle of chemotherapy was independently associated with the tomographic response, which is relevant to predict the group of patients who will not respond to treatment. The analysis of the delta was included due to its potential clinical relevance since the decision of continuing or not with more chemotherapy cycles could be made after the first chemotherapy cycle by assessing the biomarker kinetics, which can be used to early identify patients who present resistance to platinum.

Despite the main limitation is the number of patients recruited during one year at a single cancer center, this public institution is considered one of the main centers of care for cancer patients nationwide. One the other hand, the main strengths are the sample was selected with strict control, and serum levels were collected before treatment and in each cycle, being one of the studies with the highest number of patients when studying HE4 thus conveying trustable results. The authors consider that a significant association with a limited sample size suggests a strong biological effect. The results will contribute with information that can be used for extension studies to get to a more comprehensive conclusion in an effective way.

Due to the nature and design of this study, it is not possible to conclude that HE4 biomarker has higher clinical utility in comparison with CA125, nor the benefits of their combination (as the used in the Risk of Ovarian Malignancy Algorithm). Literature on the subject is scarce, particularly in Hispanic populations like ours, and most of it is retrospective and done in the adjuvant context. However, it has been shown that preoperative levels of HE4 are a predictive factor of TR. Future studies must perform comparative, and combination analyzes with the purpose of exploring the potential usefulness of HE4.

## Conclusion

Serum HE4 levels were independently associated with TR among patients with AEOC treated with NACT, also a reduction between baseline HE4 and first chemotherapy levels was also independently associated with the TR. These findings might be relevant for predicting a lack of response to treatment.

To the knowledge of the authors, this is the first report in Latin America on the kinetics of.

CA125 and HE4 as predictors of tomographic response in patients with advanced epithelial ovarian cancer treated with neoadjuvant platinum-based chemotherapy. Further study follow-up is needed to understand the impact of the biomarkers in terms of successful cytoreduction, in predicting platinum sensitivity, disease-free survival, risk to progress and overall survival.

## Supplementary Information

**Additional file 1: Table S1. **HE4 and CA125 serum levels quantified before and during the treatment with platinum-based neoadjuvant chemotherapy (n = 53).

**Additional file 2: Figure S1. **HE4 and CA125 serum levels quantified before and during the treatment with platinum-based neoadjuvant chemotherapy (n = 53).

**Additional file 3: Figure S2. **Odds ratio and 95 % confidence intervals for tomographic response versus characteristics of the patients (n = 53).

## Data Availability

The datasets used and/or analyzed during the current study are available from the corresponding author on reasonable request.

## Abbreviations

AEOC: Advanced epithelial ovarian cancer
CA125: [Serological] blood cancer antigen 125
CMIA: Chemiluminescence Microparticle Immunoassay
CTCAE 5.0: Common Terminology Criteria for Adverse Events [version] 5.0
ECOG: Eastern Cooperative Oncology Group [performance status]
FIGO: International Federation of Gynecology and Obstetrics [stage classification]
HE4: [Serological] Human epididymal protein 4
NACT: Neoadjuvant chemotherapy
NCI-Mx: National Cancerology Institute, Mexico City
OC: Ovarian cancer
PET-CT: Positron Emission Tomography-Computerized Tomography
RECIST 1.1: Response evaluation criteria in solid tumors [version] 1.1
TR: Tomographic Response
