# Supplementary material for: Kinetics of HE4 and CA125 as prognosis biomarkers during neoadjuvant chemotherapy in advanced epithelial ovarian cancer
Source: J Ovarian Res. 2021 Jul 19;14:96. doi: 10.1186/s13048-021-00845-6 (PMC8287739; doi:10.1186/s13048-021-00845-6)
Supplement: Supplementary file 1 — Additional file 1: Table S1. HE4 and CA125 serum levels quantified before and during the treatment with platinum-based neoadjuvant chemotherapy (n = 53). [file 13048_2021_845_MOESM1_ESM.docx]

**Additional file 1: Table S1.** HE4 and CA125 serum levels quantified before and during the treatment with platinum-based neoadjuvant chemotherapy (n = 53).

| **Measurement time** | **HE4 (pmol/mL)** | | **CA125 (UI/mL)** | |
| --- | --- | --- | --- | --- |
|  | **Mean (S.D.)** | **Range** | **Mean (S.D.)** | **Range** |
| **Basal** | **2150.9 (3827.4)** | **61.5 - 25596.3** | **5367.2 (7044.2)** | **28.2 - 36262.4** |
| **Cycle 1** | **853.5 (1725.2)** | **53.0 - 11764.0** | **2647.3 (2953.9)** | **33.1 - 13887.0** |
| **Cycle 2** | **525.1 (1475.9)** | **36.9 - 10782.7** | **1072.0 (1493.7)** | **7.7 - 6981.2** |
| **Cycle 3** | **408.6 (1395.0)** | **33.0 - 10216.0** | **597.8 (3827.4)** | **6.0 - 5703.0** |
