# Supplementary material for: Kinetics of HE4 and CA125 as prognosis biomarkers during neoadjuvant chemotherapy in advanced epithelial ovarian cancer
Source: J Ovarian Res. 2021 Jul 19;14:96. doi: 10.1186/s13048-021-00845-6 (PMC8287739; doi:10.1186/s13048-021-00845-6)
Supplement: Supplementary file 2 — Additional file 2: Figure S1. HE4 and CA125 serum levels quantified before and during the treatment with platinum-based neoadjuvant chemotherapy (n = 53). [file 13048_2021_845_MOESM2_ESM.docx]

**Additional file 2: Figure S1.** HE4 and CA125 serum levels quantified before and during the treatment with platinum-based neoadjuvant chemotherapy (n = 53).

**
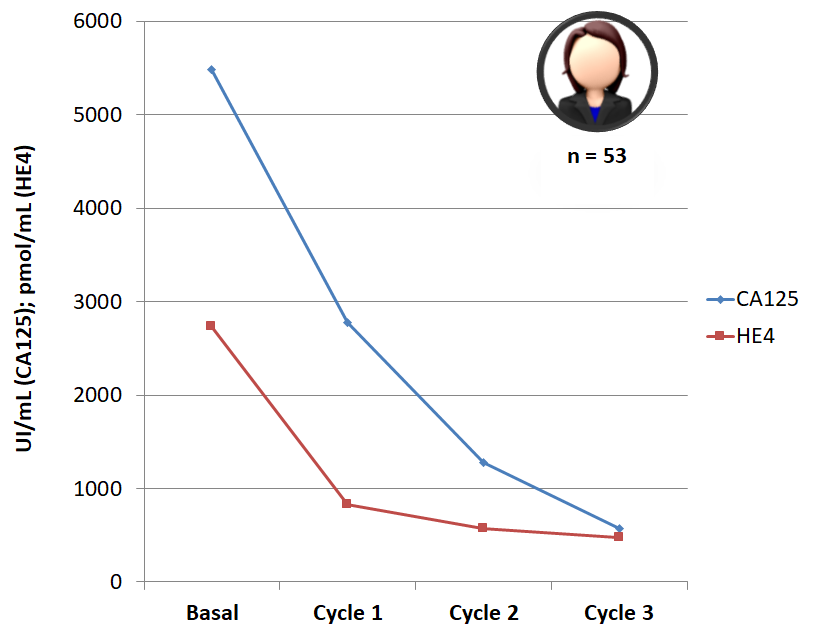
**
