# Supplementary material for: Kinetics of HE4 and CA125 as prognosis biomarkers during neoadjuvant chemotherapy in advanced epithelial ovarian cancer
Source: J Ovarian Res. 2021 Jul 19;14:96. doi: 10.1186/s13048-021-00845-6 (PMC8287739; doi:10.1186/s13048-021-00845-6)

**Additional file 3. Figure S2.** Odds ratio and 95 % confidence intervals for tomographic response versus characteristics of the patients (n = 53).


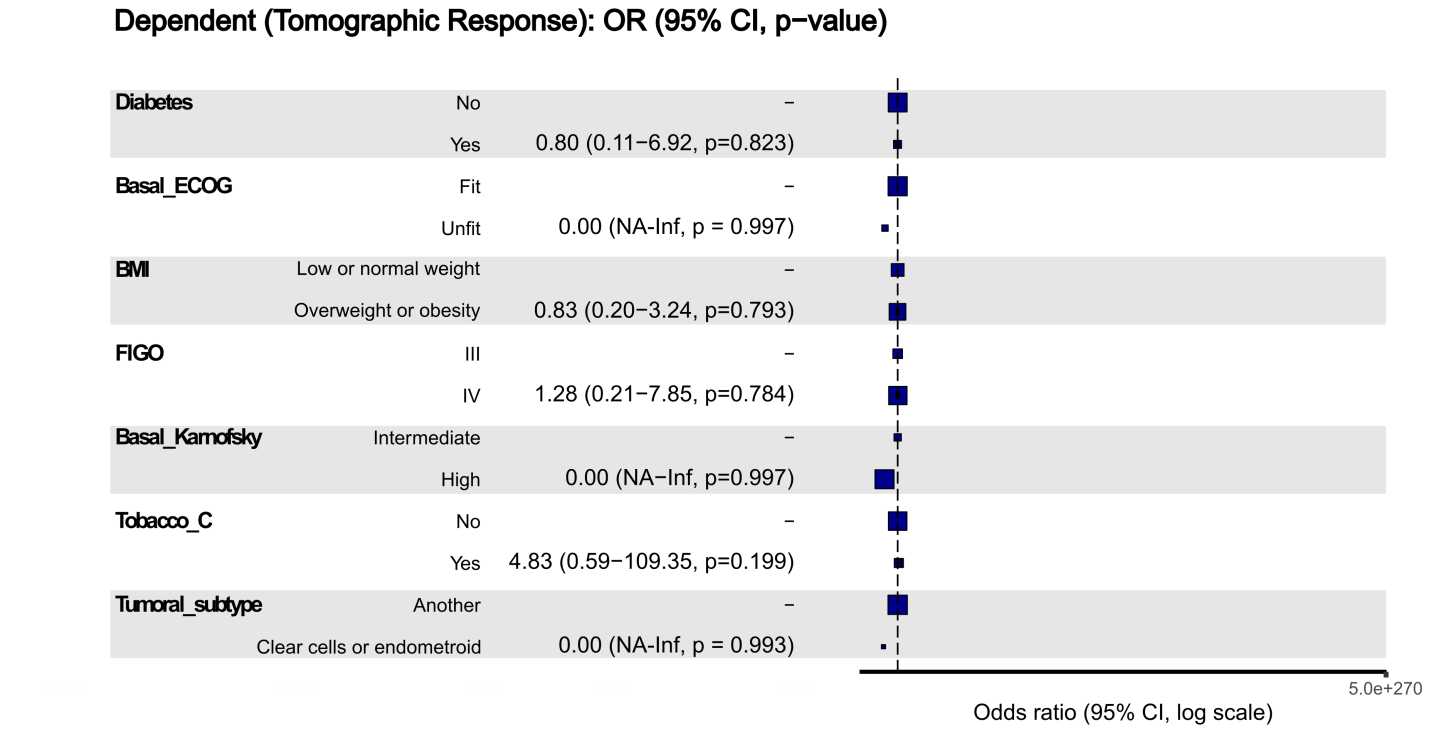

Supplement: Supplementary file 3 — Additional file 3: Figure S2. Odds ratio and 95 % confidence intervals for tomographic response versus characteristics of the patients (n = 53). [file 13048_2021_845_MOESM3_ESM.docx]
